# Thoracic Fat Pad Biopsy in Cardiac Amyloidosis: Diagnostic Yield in an Afro-Caribbean Population

**DOI:** 10.3390/jcm14051677

**Published:** 2025-03-01

**Authors:** Cedrick Mvita Bakatubia, Romain Vergier, Mathilda Simeon, Nathan Buila Bimbi, Nathan Malka, Karima Lounaci, Maria Herrera Bethencourt, Karim Fard, Arnt Kristen, Rishika Banydeen, Astrid Monfort, Jocelyn Inamo, Andreas Müssigbrodt

**Affiliations:** 1 Department of Cardiology, CHU Martinique (University Hospital of Martinique), 97200 Fort de France, France; mvitacedric1@gmail.com (C.M.B.); romain.vergier@chu-martinique.fr (R.V.); karima.lounaci@chu-martinique.fr (K.L.); maria.herrerabethencourt@gmail.com (M.H.B.); astrid.monfort@chu-martinique.fr (A.M.); jocelyn.inamo@chu-martinique.fr (J.I.); 2 Department of Cardiology, University Hospital of Kinshasa, Kinshasa 01302, Democratic Republic of the Congo; drnathanbuila@gmail.com; 3 Department of Pathology, CHU Martinique (University Hospital of Martinique), 97200 Fort de France, France; mathilda.simeon@live.fr; 4 Clinique Ambroise Paré–Hartmann, 92200 Neuilly-sur-Seine, France; docteur.malka@hotmail.com; 5 Hôtel-Dieu, AP-HP, 75004 Paris, France; 6 Caribbean Institute of Nuclear Imaging (ICIN), CHU Martinique (University Hospital of Martinique), 97200 Fort de France, France; karim.fard@chu-martinique.fr; 7 Department of Cardiology, Angiology, Respiratory Medicine, Medical University of Heidelberg, 69120 Heidelberg, Germany; kristen@kardio-darmstadt.de; 8 Clinical Research Unit, Department of Critical Care and Emergency Medicine, CHU Martinique (University Hospital of Martinique), 97200 Fort de France, France; rishika.banydeen@chu-martinique.fr; 9 Cardiovascular Research Team (UR5_3 PC2E), University of the French West Indies (Université des Antilles), 97200 Fort de France, France

**Keywords:** amyloidosis, ATTR, cardiac, scintigraphy, fat pad, biopsy, pacemaker

## Abstract

**Background/Objectives**: Cardiac amyloidosis (CA) is associated with amyloid infiltration of the extra-cardiac tissue, which may occur in the early stages of the disease. This study evaluates the diagnostic utility of thoracic fat pad biopsy obtained during a pacemaker or ICD implantation as an alternative to the standard diagnostic criteria for systemic amyloidosis. **Methods**: This exploratory, retrospective study included 27 patients with suspected or diagnosed CA who underwent pacemaker or defibrillator therapy. **Results**: Of these, 16 patients were confirmed to have CA (15 with technetium-labeled bisphosphonate bone scintigraphy and 1 with protein electrophoresis and echocardiographic findings) while 11 were confirmed to be CA-negative. The thoracic fat pad biopsy demonstrated a specificity of 100% but a sensitivity of only 31%. Among patients with transthyretin (ATTR)-CA, the sensitivity remained similarly low, at 27%. These results are consistent with prior findings on abdominal fat pad biopsy in ATTR-CA, highlighting the limited diagnostic yield of this method. **Conclusions**: Thoracic fat pad biopsy cannot be recommended as a standard diagnostic tool for CA, particularly in ATTR-CA, due to its poor sensitivity. However, in AL (amyloid light-chain) amyloidosis, this minimally invasive procedure may aid diagnosis without additional invasive interventions.

## 1. Introduction

Amyloidosis is a severe condition resulting from the systemic accumulation of amyloid protein deposits in various tissues, leading to structural and functional abnormalities. It is primarily associated with cardiac and neurological manifestations, with its cardiac and renal forms posing significant threats to health and life. Cardiac amyloidosis (CA) primarily manifests as restrictive cardiomyopathy, which can result in heart failure and arrhythmias. Amyloidosis can arise from various types of amyloid proteins, but in Europe, the most prevalent forms are amyloid light-chain (AL) and transthyretin (ATTR) amyloidosis [1,2].

The hereditary form of ATTR cardiac amyloidosis (ATTRv-CA) disproportionately afflicts the black Afro-Caribbean population, who, when compared to mainly whites with wild-type transthyretin amyloidosis (wt-ATTR-CA), a phenotypically similar condition, present with more advanced disease despite genetic testing that may permit early identification [3].

Patients with ATTR amyloidosis may experience musculoskeletal symptoms 5 to 15 years prior to signs or symptoms of systemic disease [4,5,6]. Carpal tunnel syndrome, the most prevalent non-cardiac symptom in patients with ATTR-CA, often appears years before a formal diagnosis of either wt-ATTR-CA or hereditary ATTRv-CA [4]. ATTR amyloid deposits could be identified in various tissues of patients with amyloidosis removed during orthopedic procedures, including carpal tunnel syndrome, rotator cuff tears, and lumbar canal stenosis [7].

**Figure 1:**
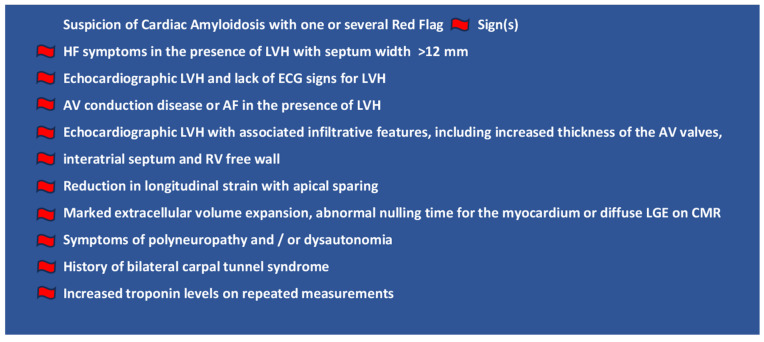
Possible clinical signs that should raise the suspicion of cardiac amyloidosis (CA). HF—heart failure; LVH—left ventricular hypertrophy; AV—atrioventricular; AF—atrial fibrillation; RV—Right Ventricular; LGE—Late Gadolinium Enhancement; CMR—cardiac magnetic resonance tomography.

Identifying the early manifestations of CA could potentially shorten the diagnostic delay, enabling early therapeutic intervention [2,8,9]. Among these early signs, conduction disorders or other arrhythmias necessitating the implantation of pacemakers or defibrillators may be early manifestations of CA. Early signs of CA, often referred to as “red flag” symptoms (Figure 1), should prompt suspicion of CA and initiate further diagnostic evaluation [1,6,10].

Echocardiography, with its advanced modalities such as strain imaging, speckle tracking, and myocardial work analysis, plays a crucial role in diagnosing CA and differentiating it from other cardiac conditions associated with left ventricular hypertrophy (LVH), such as hypertrophic cardiomyopathy [11,12,13].

Definitive diagnosis relies on histopathological examination of the affected tissue, which involves biopsy procedures that can be particularly challenging and pose risks, especially in the cardiac and renal forms of the disease. Historically, conclusive diagnosis of amyloidosis has been made through tissue biopsy stained with Congo red [14]. When observed under polarized light, this reveals the characteristic green birefringence of amyloid deposits, confirming the presence of the disease [14]. Histological confirmation is still required in cases where both bone scintigraphy (indicating ATTR-CA) and tests for monoclonal protein (indicating potential AL amyloidosis) show abnormalities or are inconclusive [14]. It is also reasonable to verify and classify amyloid deposits through immunohistochemistry or mass spectrometry [15]. Mass spectrometry is the currently preferred method for amyloid typing, as immunohistochemistry results are often subtle and can be misinterpreted without significant expertise [15].

Bone scintigraphy, particularly using 99m-technetium-labeled bisphosphonates, has emerged as a valuable diagnostic tool for ATTR-CA [16]. This imaging technique can non-invasively detect myocardial uptake of amyloid deposits, offering high sensitivity and specificity in distinguishing ATTR-CA from other forms, such as AL-CA, after exclusion of monoclonal gammopathy [16]. The limited availability of bone scintigraphy in less-equipped healthcare settings may pose a significant barrier to the timely and accurate diagnosis of cardiac ATTR amyloidosis.

Both ventricular and supraventricular arrhythmias are frequently observed in CA, particularly in ATTR-CA, with their occurrence closely linked to disease progression [17]. The prognostic significance of sustained and non-sustained ventricular tachycardias remains uncertain, and there are currently no clear guidelines for the use of implantable cardiac defibrillators (ICDs) in these patients [17]. Atrial fibrillation is the most common supraventricular arrhythmia, affecting up to 88% of individuals with ATTR-CA [17]. Anticoagulation should be considered regardless of the CHADsVA score [18]. Conduction abnormalities and bradyarrhythmias are also prevalent in ATTR-CA, with up to 40% of patients requiring pacemaker implantation [17].

The implantation of pacemakers involves creating a pre-pectoral pocket between the superficial skin plane and the deeper muscular plane. One case report described amyloid deposits within the pacemaker pocket of a patient who had already been diagnosed with ATTRv-CA [19].

The potential for amyloid infiltration in the pacemaker or ICD pocket during the early stages of the disease has not been systematically investigated and warrants further study. As conduction abnormalities and other arrhythmias are linked to amyloidosis, the presence of peripheral deposits in the pacemaker or ICD pocket would not be unexpected.

The current study aims to assess the diagnostic utility of thoracic fat pad biopsy obtained from the pacemaker or ICD pocket as a potential alternative biopsy site for patients with suspicion of systemic amyloidosis. This exploratory, descriptive, hypothesis-generating study will compare the sensitivity and specificity of thoracic fat pad biopsy against standard diagnostic approaches for detecting CA, providing insights into the value of histopathological analysis of fat pad samples collected during pacemaker or ICD implantation. By exploring this previously unstudied method, we aim to determine the clinical yield of an accessible diagnostic tool that could facilitate earlier intervention and improve the management of patients with CA.

## 2. Methods

Patients with suspicion or diagnosis of CA and indications of pacemaker or ICD implantation or generator change, admitted in our electrophysiology (EP) lab between October 2020 and August 2024, were included in this exploratory, observational, retrospective single-center study.

**Figure 2:**
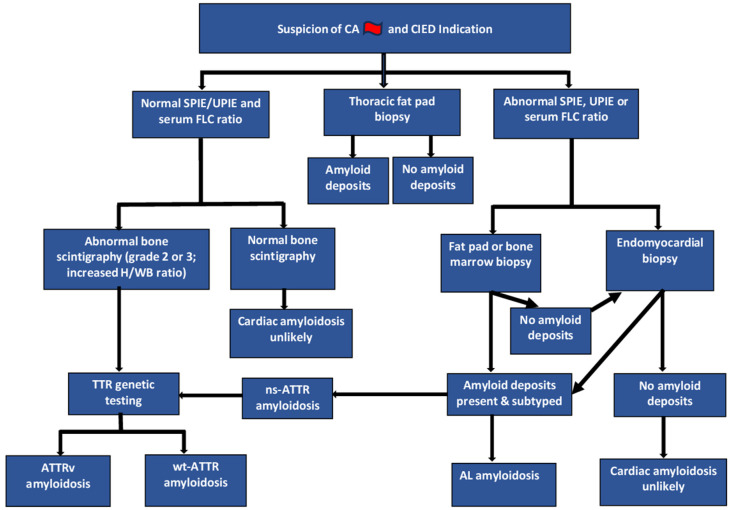
Algorithm for the evaluation of suspected cardiac amyloidosis at the University Hospital Martinique with added thoracic fat pad biopsy during pacemaker implantation. CA—cardiac amyloidosis; CIED—cardiac implantable electronic device; TTR—transthyretin; AL—amyloid light-chain; FLC—free light chain; GI—gastrointestinal; SPIE—serum protein electrophoresis with immunofixation; UPIE—urine protein electrophoresis with immunofixation; H/WB—heart-to-whole body ratio, ns-ATTR—non-specified ATTR; ATTRv—hereditary (variant) ATTR; wt-ATTR—wild type (senile) ATTR. Algorithm modified and adapted after [10,22].

The initial assessment included “red flag” criteria, such as heart failure (HF) symptoms in the presence of LVH (Figure 1). Eligible patients were selected if (1) the “red flag” criteria indicated a possible yet undiagnosed CA or if CA was already diagnosed and (2) the patients had indications of pacemaker or ICD implantation or generator change. The patients who fulfilled these criteria were consecutively included. The indication of pacemaker and ICD therapy followed current guidelines [20,21]. The diagnosis of CA was established with the current gold-standard diagnostic tools (Figure 2) [22]. This analysis involved SPIE (serum protein electrophoresis with immunofixation), FLC (free light chain) testing, and UPIE (urine protein electrophoresis with immunofixation). Patients with suspected CA and negative or non-conclusive SPIE underwent bone scintigraphy. In our center, we use technetium-99m-labeled hydroxymethylene diphosphonate (99mTc-HMDP) for bone scintigraphy. 99mTc-HMDP scintigraphy involves administering 10 to 25 mCi of radiotracer intravenously, followed by planar and single-photon emission computed-tomography (SPECT) imaging, typically performed 1 or 3 h later. The degree of myocardial tracer uptake on planar imaging can be assessed using both qualitative and quantitative grading systems. Qualitative grading compares heart uptake with rib-bone uptake, with grade 0 indicating no uptake, grade 1 representing mild uptake (less than rib), grade 2 showing moderate uptake (equal to rib), and grade 3 indicating severe uptake (greater than rib). Quantitative assessment relies on the heart-to-whole body (H/WB) ratio [16]. Genetic sequencing allows the distinguishing of ATTRv from wt-ATTR.

Thoracic fat pad biopsy was integrated into the diagnostic workflow during pacemaker or ICD implantation (Figure 2). The sequence and timing of the diagnostic tests were decided upon at the discretion of the physician in charge, depending on clinical needs. All implantation procedures were performed under local anesthesia by two experienced electrophysiologists (A.M., R.V.) or by a fellow electrophysiologist (C.M.).

All histopathological samples were examined by experienced cytopathologists. Following fixation in 4% formalin, the samples were embedded in cell blocks and stained with standard HE (Hematoxylin–Eosin). Sections were cut at a thickness of 3 microns, with six levels of cuts prepared for each sample. Congo red staining was performed on the frozen cell block samples, which were sectioned at 8 microns. The slides were counterstained with HE for four minutes, pretreated in an alkaline salt solution for 20 min, and subsequently stained with Congo red for an additional 20 min. A positive result was defined by the presence of apple-green birefringence under polarized light, observed around the fat cells or within the vessel walls. In contrast, linear white birefringence, characteristic of collagen, was not considered a diagnostic for amyloidosis. We utilized a slide scanner (Leica Aperio GT 450DX) to digitize the HE- and Congo red-stained slides, which were then processed through the Calopix platform for visualization. For the birefringence imaging, photographs were manually captured (Nikon Eclipse Ni-U).

The patient and procedure selection were non-randomized. The study data (patient and procedure characteristics) were retrospectively collected. Power calculations were not performed in this exploratory, retrospective study, as it is the first to evaluate this novel fat biopsy site, with no prior data to guide sample size estimation. Additionally, the limited number of available patients further precluded power calculations. Statistical analysis was performed with JASP Team (2024, Version 0.19.1). Means and standard deviations were reported for the quantitative variables, while the categorical variables were presented as absolute values and percentages. The following tests were accordingly used for group comparisons: the t-test, the Wilcoxon–Mann–Whitney test, the Chi-square test, and Fisher’s exact test. Statistical significance was set at *p* < 0.05. The sensitivity was calculated from the number of true positives divided by the sum of the number of true positives and the number of false negatives. The specificity was calculated from the number of true negatives divided by the sum of the number of true negatives and the number of false positives.

All patients were managed in accordance with the amended Declaration of Helsinki (https://www.wma.net/what-we-do/medical-ethics/declaration-of-helsinki/, accessed on 18 December 2024). Written informed consent from the patients or patients’ legal guardians/next of kin was not required to participate in this study in accordance with current legislation and institutional requirements. All patients provided written informed consent prior to the implantation procedure. This study was approved by the Institutional Review Board of the University Hospital of Martinique (reference number 2025/009).

## 3. Results

A total of 27 patients were included in this study. Of these, 16 were diagnosed with CA, while 11 did not have CA based on the standard diagnostic criteria (Table 1 and Table S1, Supplymentary Materials). Among the 16 CA patients, 15 (93.8%) had ATTR-CA and 1 (6.3%) had AL-CA (Table 1 and Table S1, Supplymentary Materials). Of the ATTR-CA patients, eight individuals were found to carry a heterozygous mutation for the amyloidogenic allele where isoleucine substituted for valine at codon position 142 (122 of the mature protein) (p.Val142Ile), leading to the diagnosis of hereditary ATTR-CA (ATTRv-CA). The genetic testing was negative in three patients, leading to the diagnosis of wild-type ATTR-CA (wt-ATTR-CA). Genetic testing was not performed in four patients with ATTR-CA. As no definitive diagnosis of ATTRv-CA or wt-ATTR-CA could be made, we defined those patients as non-specified ATTR-CA (ns-ATTR-CA) (Table 1 and Table S1, Supplymentary Materials).

No complications related to the intervention occurred. The baseline characteristics were similar between the patients with and without CA, with no significant differences in age, sex, BMI, QRS width, LVEF, renal function, or BNP levels. However, the patients with CA had higher baseline heart rates, less arterial hypertension, lower LVEFs, and greater septal thickness (Table 1). The majority of the patients without CA (81.8%) had arterial hypertension and were diagnosed with hypertensive cardiomyopathy. The indication for pacemaker therapy was atrioventricular conduction disease, in 13 patients (81.3%) in the amyloidosis group and in 10 patients (90.9%) in the group without amyloidosis. Atrioventricular (AV) conduction disease was defined as second-degree AV block, complete AV block, alternating bundle branch block, bi-fascicular block, or AF with bradyarrhythmia. One patient (9.1%) in the group without amyloidosis was diagnosed with symptomatic sick sinus syndrome. Three patients received ICDs. The ICD indications were an LVEF of 30% and non-sustained ventricular tachycardia in a 63-year-old patient with wt-ATTR-CA, an LVEF of 30% in a 64-year-old patient with ATTRv-CA, and an LVEF of 28% in a 56-year-old patient with AL-CA.

**Figure 3:**
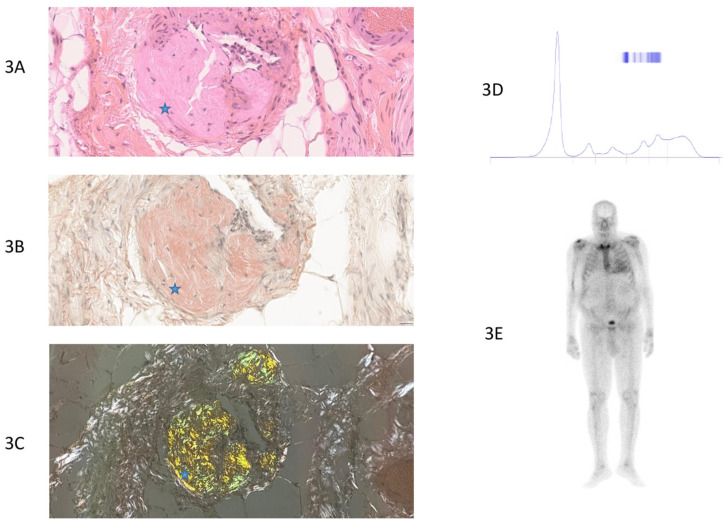
True-positive case of a patient with hereditary transthyretin ATTRv (p.Val142Ile) cardiac amyloidosis (patient number 9, Table S1, Supplementary Materials). (**3A**) Standard HE (Hematoxylin–Eosin) staining of thoracic fat pad biopsy with 40× magnification. Fibro-adipose tissue without atypia, showing amyloid deposits in the vascular walls as amorphous eosinophilic extracellular deposits. Blue star: amyloid deposits. (**3B**) Congo red staining of thoracic fat pad biopsy, 40× magnification. Fibro-adipose tissue without atypia, showing amyloid deposits stained with Congo red. Blue star: amyloid deposits. (**3C**) Amyloid deposits displaying yellow–green birefringence under polarized light (on Congo red-stained slides). Blue star: amyloid deposits. (**3D**) Normal protein electrophoresis without monoclonal peak. (**3E**) Bone scintigraphy: grade 3 uptake in the cardiac area, suggestive of transthyretin cardiac amyloidosis. Diffuse uptake in soft tissues.

**Figure 4:**
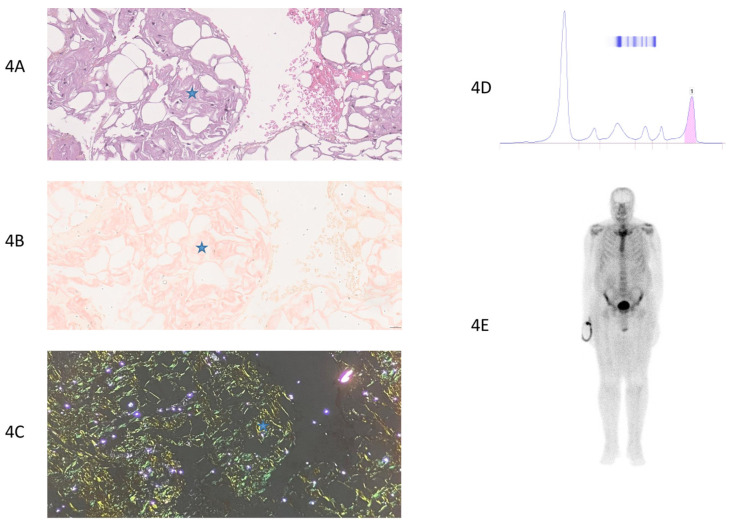
True-positive case of a patient with AL cardiac amyloidosis (patient number 27, Table S1, Supplementary Materials). (**4A**) Standard HE (Hematoxylin–Eosin) staining of thoracic fat pad biopsy with 40× magnification. Fibro-adipose tissue without atypia, showing amyloid deposits as amorphous eosinophilic extracellular deposits. Blue star: amyloid deposits. (**4B**) Congo red staining of thoracic fat pad biopsy, 40× magnification. Fibro-adipose tissue without atypia, showing amyloid deposits stained with Congo red. Blue star: amyloid deposits. (**4C**) Amyloid deposits displaying yellow–green birefringence under polarized light (on Congo red-stained slides). Blue star: amyloid deposits. (**4D**) Protein electrophoresis: monoclonal peak. (**4E**) Bone scintigraphy: no uptake observed in the cardiac area, grade 0 uptake. Moderate diffuse uptake in extra-cardiac tissue.

**Figure 5:**
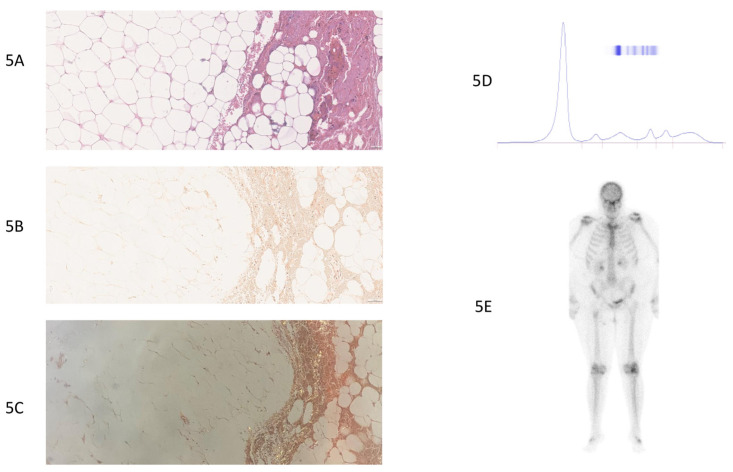
True-negative case of a patient without cardiac amyloidosis (patient number 15, Table S1, Supplementary Materials). (**5A**) Standard HE (Hematoxylin–Eosin) staining of thoracic fat pad biopsy, 20× magnification: fibro-adipose tissue without amyloid deposits. (**5B**) Congo red staining, 20× magnification. Fibro-adipose tissue without amyloid deposits. (**5C**) Absence of yellow–green birefringence under polarized light. (**5D**) Protein electrophoresis: normal, without monoclonal peak. (**5E**) Bone scintigraphy: no uptake in the cardiac area, grade 0. No suspicious uptake foci detected on the whole-body scan. No scintigraphic evidence of transthyretin cardiac amyloidosis.

**Figure 6:**
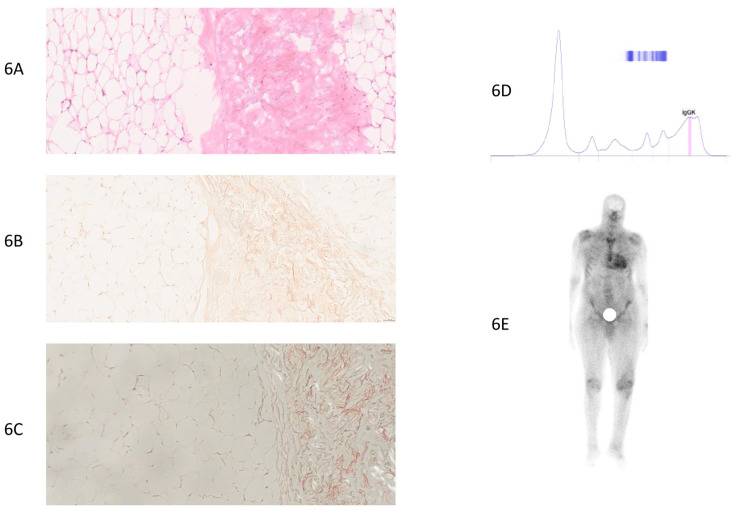
False-negative case of a patient with hereditary transthyretin ATTRv (p.Val142Ile) cardiac amyloidosis (patient number 23, Table S1, Supplymentary materials). (**6A**) Standard HE (Hematoxylin–Eosin) staining, 20× magnification. Fibro-adipose tissue without atypia, no amyloid deposits. (**6B**) Congo red staining, 20× magnification. Adipose tissue without atypia, no amyloid deposits. (**6C**) Absence of amyloid deposits (yellow–green) under polarized light (on slide stained with Congo red). (**6D**) Protein electrophoresis: monoclonal peak (IgGkappa). The hematological evaluation, including serum protein electrophoresis, immunofixation, serum-free light-chain analysis, Bence-Jones protein assessment, clinical evaluation, bone marrow biopsy, and imaging, excluded plasmacytoma and confirmed MGUS (monoclonal gammopathy of undetermined significance). The diagnosis of hereditary ATTRv (p.Val142Ile) cardiac amyloidosis was established through positive bone scintigraphy and supported by genetic testing. (**6E**) Bone scintigraphy: intense uptake in the cardiac area, with intensity greater than the costal framework, grade 3 uptake, suggestive of transthyretin cardiac amyloidosis. Diffuse soft tissue uptake. Degenerative uptake in both knees.

The histopathological analysis showed a specificity of 100% and a sensitivity of 31.3% in the studied cohort (Table 2). Figure 3, Figure 4, Figure 5 and Figure 6 provide examples of two true-positive cases, one true-negative case, and one false-negative case. With no false-positive results, the positive predictive value (PPV) based on the cohort data was 100%, indicating that all the positive biopsy results were true positives for CA. When accounting for the cohort prevalence of 59.3%, the PPV was calculated to be 18.5%, reflecting the diagnostic performance in the context of this specific population. The negative predictive value (NPV) was 50%, indicating that half of the negative biopsies were false negatives (Table 2).

As previous studies have demonstrated a lower sensitivity of abdominal fat pad biopsy in patients with ATTR-CA compared with those with AL-CA, we analyzed the sensitivity and specificity for ATTR-CA separately, excluding the patient with AL-CA. The sensitivity was again found to be poor, at 26.7%, with a specificity of 100% (Table 3).

In our study, we did not routinely perform biopsies from the abdominal fat pad. Therefore, the fat pad biopsies from only eight patients could be compared with the results from the thoracic fat pad biopsies. Three patients with amyloid evidence in the abdominal fat pad biopsy had also amyloid evidence in the thoracic fat pad biopsy. One of them was diagnosed with ns-ATTR-CA (grade 3 myocardial uptake), one with ATTRv-CA (grade 4 uptake), and one with AL-CA. Five patients without amyloid evidence in the abdominal fat pad biopsy also had no amyloid evidence in the thoracic fat pad biopsy (*p* = 0.018). Among the five patients without amyloid evidence in their abdominal fat pad biopsies, two had ATTRv-CA with grade 2 and 3 myocardial uptake (false-negative cases), respectively, while three showed no evidence of CA (true-negative cases) based on the standard diagnostic criteria (*p* = 0.196). In this group of eight patients with thoracic and abdominal fat pad biopsies, we found an excellent correlation with identical results from both anatomical sites (*p* = 0.018). In this small group, the thoracic and abdominal fat pad biopsies detected only three out of five cases with CA (sensitivity 60%, specificity 100%, *p* = 0.196). Seven patients also received salivary gland biopsy. We found three true-positive cases and four false-negative cases compared to the gold-standard diagnostic.

## 4. Discussion

The prevalence of amyloidosis in the general population of Martinique is unknown. However, deducing from the known 3.43% prevalence of the p.Val142Ile variant in African Americans and assuming disease manifestation in 10–15% of carriers, one could estimate a prevalence of manifest ATTRv-CA between 0.3% and 0.5% in Martinique, a Caribbean island where approximately 90% of the population is of African descent [3,23]. The prevalence of manifest amyloidosis in African countries is also unknown but is likely to increase in the future due to the increasing life expectancy [24]. The limited availability of bone scintigraphy in under-resourced healthcare settings may present a substantial challenge to the timely and accurate diagnosis of cardiac ATTR amyloidosis. While the cost of bone scintigraphy ranges from 400 to 1000 EUR, histological diagnostics can be performed at approximately 10% of this expense. This underscores the critical need for accessible and cost-effective diagnostic tools.

In patients with clinical amyloidosis (cardiac or neurological), the diagnostic yields of peripheral biopsies vary across studies. The specificity rates are excellent, consistently reaching 100%, while the sensitivity rates show more variation. Depending on the etiology and in the most favorable studies, abdominal fat biopsies demonstrate a sensitivity from 58 to 95% and rectal mucosal biopsies range from 74 to 95% [25,26,27]. Biopsies of the accessory salivary glands exhibit sensitivities between 45 and 61%, and renal biopsies range from 85 to 100% [28,29,30]. Additionally, some studies report a very high diagnostic yield from endomyocardial biopsies in characteristic cardiac amyloidosis cases [14,31].

The patients with CA demonstrated—when compared with the patients without CA—a higher baseline heart rate, less arterial hypertension, lower LVEFs, and greater septal thickness (Table 1). These findings can be explained, as patients with CA are more likely to be diagnosed with atrial fibrillation, dysautonomia, and diastolic HF, all leading to increased heart rates. Amyloid deposits may also lead to lower LVEFs and greater septal thickness when compared to patients without amyloid deposits. The patients with CA also presented less arterial hypertension than the comparison group that mainly consisted of patients with LVH due to hypertensive cardiopathy.

In this exploratory, descriptive analysis, the histopathological analysis of a thoracic fat pad sample from the pacemaker or ICD pocket could diagnose only five of sixteen patients with CA. In total, 11 of the 16 patients with diagnoses of CA had no amyloid evidence in the histopathological analysis of the thoracic fat pad biopsy. Thus, the sensitivity of the histopathological analysis to diagnose CA was poor, with only 31%. Noteworthily, there were no false-positive findings.

No correlation was observed between the myocardial uptake in bone scintigraphy and the thoracic fat pad biopsy results. Even though no wt-ATTR-CA was among the cases with positive amyloid findings in the thoracic fat pad sample, no reasonable conclusions can be drawn due to the small sample size.

Although this observation should be interpreted with great caution due to the small number of cases, we found, in a group of eight patients with thoracic and abdominal fat pad biopsies, an excellent correlation, with identical results from both anatomical sites (*p* = 0.018).

The limited sensitivity of 31% of the thoracic fat pad biopsy in our study for 94% of the patients with ATTR-CA is similar to findings of previous studies on abdominal fat pad aspiration. Those studies demonstrated that the sensitivity of abdominal fat pad aspiration greatly varies depending on the type of amyloid protein. The sensitivity for the diagnosis of ATTR amyloidosis using abdominal fat pad aspiration or biopsy depends greatly on the type of amyloid protein. It was 15% for wt-ATTR-CA, 39% for ns-ATTR-CA, and 45% for ATTRv-CA, whereas the sensitivity in patients with AL-CA was up to 84–90% [32,33]. Possible explanations for the limited and variable sensitivity of the fat pad aspiration may be the uneven distribution of amyloid deposits in different tissues and the limitations of current histopathological methods to detect minimal quantities of amyloid deposits.

The limitations of this study include the small sample size (n = 27), the retrospective single-center design, the lack of power calculations due to the exploratory nature of the research, and the limited imaging parameters (i.e., CMR), which may limit the generalizability of the findings to broader populations.

## 5. Conclusions

Our initial hypothesis that cardiac amyloidosis (CA) could be reliably diagnosed through histopathological analysis of thoracic fat pad samples obtained from pacemakers or ICD pockets was not confirmed. In this study involving an Afro-Caribbean population, with 94% diagnosed with ATTR-CA, thoracic fat pad biopsy demonstrated a sensitivity of 31% and a specificity of 100% compared to protein electrophoresis and technetium-labeled bisphosphonate bone scintigraphy as standard diagnostic tools. Given its low diagnostic yield, histopathological analysis of thoracic fat pad biopsies cannot currently be recommended as a standard diagnostic tool for ATTR-CA. However, it may still hold significant value in diagnosing AL-CA, potentially obviating the need for further invasive procedures. This study underscores the importance of utilizing the established standard diagnostic criteria for CA while also highlighting the need for less expensive and more accessible diagnostic methods, especially in resource-limited settings.

## Figures and Tables

**Table 1:** Baseline parameters comparing patients with and without cardiac amyloidosis. Quantitative variables are presented as means ± standard deviation and qualitative variables as absolute numbers, n (%); level of significance: *p* < 0.05. bpm—beats per minute; BMI—body mass index; ATTR-CA—ATTR cardiac amyloidosis; AV—atrioventricular; ICD—implantable cardioverter defibrillator; LVEF—left ventricular ejection fraction; GFR—glomerular filtration rate; CKD—chronic kidney disease with GFR < 60 mL/min; BNP—brain natriuretic peptide.

	Amyloidosis (n = 16)	No Amyloidosis (n = 11)	*p*-Value
**Age (years ± SD)**	76.5 ± 9	77.1 ± 10.2	0.870
**Male sex, n (%)**	15 (93.8)	7 (63.6)	0.125
**BMI (kg/m^2^ ± SD)**	25.1 ± 4.2	28.5 ± 6.1	0.118
**ATTR-CA, n (%)**	15 (93.8)	0 (0%)	0.001
**Baseline heart rate (bpm ± SD)**	59 ± 25	41 ± 8	0.026
**Atrial fibrillation, n (%)**	6 (37.5%)	3 (33.3)	0.692
**Sick sinus syndrome, n (%)**	0 (0)	1 (9.1)	0.407
**AV conduction disease, n (%)**	13 (81.3)	10 (90.9)	0.624
**Pacemaker, n (%)**	13 (81.3)	11 (100)	0.248
**ICD n (%)**	3 (18.8)	0	0.248
**QRS width (ms ± SD)**	127 ± 37	110 ± 34	0.422
**LVEF (% ± SD)**	46 ± 11	55 ± 9	0.053
**Septum (mm ± SD)**	15 ± 3	12 ± 2	0.027
**Arterial hypertension, n (%)**	4 (25)	9 (81.8)	0.006
**GFR (mL/min ± SD)**	64 ± 25	73 ± 34	0.461
**CKD n (%)**	7 (43.8)	4 (36.4)	1.000
**BNP (pg/mL ± SD)**	465.3	574.4	0.676

**Table 2:** Cross table for CA (AL-CA and ATTR-CA): sensitivity, 31.3%; specificity, 100%; positive predictive value, 18.5%; negative predictive value, 50%; Fisher’s exact test: *p* = 0.06. CA—cardiac amyloidosis.

	CA	No CA
**Biopsy positive with evidence of amyloid deposits n (%)**	5 (31.3)	0 (0)
**Biopsy negative without evidence of amyloid deposits n (%)**	11 (68.7)	11 (100)

**Table 3:** Cross table for ATTR-CA: sensitivity, 26.7%; specificity, 100%; Fisher’s exact test: *p* = 0.113. ATTR-CA—transthyretin cardiac amyloidosis.

	ATTR-CA	No ATTR-CA
**Biopsy positive with evidence of amyloid deposits, n (%)**	4 (26.7)	0 (0)
**Biopsy negative without evidence of amyloid deposits, n (%)**	11 (73.3)	11 (100)

## Data Availability

The data that support the findings of this study are available from the corresponding author (AM) upon reasonable request.

## Supplementary Materials

The following supporting information can be downloaded at: https://www.mdpi.com/article/10.3390/jcm14051677/s1, Table S1: Patient characteristics of all patients with definite diagnosis and thoracic fat pad biopsy.
