# Supplementary material for: Thoracic Fat Pad Biopsy in Cardiac Amyloidosis: Diagnostic Yield in an Afro-Caribbean Population
Source: J Clin Med. 2025 Mar 1;14(5):1677. doi: 10.3390/jcm14051677 (PMC11900135; doi:10.3390/jcm14051677)
Supplement: Supplementary file 1 [file jcm-14-01677-s001.zip › jcm-3412653-supplementary.pdf]

Table S1 (supplementary material): Patient characteristics of all patients with definite diagnosis and thoracic fat pad biopsy. AL amyloid light-chain; ATTR transthyretin amyloidosis; CA cardiac amyloidosis; ATTRv-CA hereditary (variant) ATTR-CA; wt-ATTR-CA wild-type ATTR-CA; ns-ATTR-CA non-specified ATTR-CA (without genetic diagnostic); p.Val142Ile heterozygous mutation for the amyloidogenic allele where isoleucine substitutes for valine at codon position 142 (122 of the mature protein); MGUS monoclonal gammopathy of undetermined significance.

Number	Diagnosis	Serum electrophoresis	Salivary glands biopsy	Abdominal fat pad biopsy	Thoracic fat pad biopsy	Bone scintigraphy	Result
1	No CA	Normal	None	None	Negative	Negative	True negative
2	ATTRv-CA (p.Val142Ile)	Normal	None	None	Negative	Positive, Grade 3 uptake	False negative
3	No CA	Normal	None	None	Negative	Negative	True negative
4	ns-ATTR-CA	Normal	None	None	Negative	Positive, Grade 2 uptake	False negative
5	ATTRv-CA (p.Val142Ile)	Normal	Positive	Positive	Positive	Positive, Grade 4 uptake	True positive
6	No CA	Normal	None	Negative	Negative	Negative	True negative
7	No CA	Normal	None	None	Negative	Negative	True negative
8	ns-ATTR-CA	Normal	None	Positive	Positive	Positive, Grade 3 uptake	True positive
9	ATTRv-CA (p.Val142Ile)	Normal	Negative	None	Positive	Positive, Grade 3 uptake	True positive
10	No CA	Normal	None	Negative	Negative	Negative	True negative
11	No CA	Normal	None	None	Negative	Negative	True negative
12	No CA	Normal	None	None	Negative	Negative	True negative
13	ATTRv-CA (p.Val142Ile)	Monoclonal peak (MGUS)	Positive	Negative	Negative	Positive, Grade 3 uptake	False negative
14	No CA	Normal	None	None	Negative	Negative	True negative
15	No CA	Normal	None	Negative	Negative	Negative	True negative
16	No CA	Normal	None	None	Negative	Negative	True negative
17	No CA	Normal	None	None	Negative	Negative	True negative
18	ATTRv-CA (p.Val142Ile)	Normal	None	None	Negative	Positive, Grade 3 uptake	False negative
19	ns-ATTR-CA	Normal	None	None	Negative	Positive Grade 2 uptake	False negative
20	ns-ATTR-CA	Normal	Negative	None	Negative	Positive, Grade 2 uptake	False negative
21	wt-ATTR-CA	Normal	Negative	None	Negative	Positive, Grade 2 uptake	False negative
22	wt-ATTR-CA	Normal	None	None	Negative	Positive, Grade 3 uptake	False negative

23	ATTRv-CA (p.Val142Ile)	Monoclonal peak (MGUS)	Negative	Negative	Negative	Positive, Grade 3 uptake	False negative
24	ATTRv-CA (p.Val142Ile)	Normal	None	None	Positive	Positive, Grade 2 uptake	True positive
25	ATTRv-CA (p.Val142Ile)	Normal	Negative	None	Negative	Positive, Grade 2 uptake	False negative
26	wt-ATTR-CA	Normal	None	None	Negative	Positive, Grade 3 uptake	False negative
27	AL-CA	Monoclonal peak	Positive	Positive	Positive	Negative	True positive
